# Body composition assessment with ultrasound muscle measurement: optimization through the use of semi-automated tools in colorectal cancer

**DOI:** 10.3389/fnut.2024.1372816

**Published:** 2024-04-17

**Authors:** Fiorella Palmas, Fernanda Mucarzel, Marta Ricart, Amador Lluch, Alba Zabalegui, Jose Melian, Raul Guerra, Aitor Rodriguez, Nuria Roson, Andreea Ciudin, Rosa Burgos

**Affiliations:** ^1^Endocrinology and Nutrition Department, Hospital Universitari Vall D’Hebron, Barcelona, Spain; ^2^Centro de investigación Biomédica en Red de Diabetes y Enfermedades Metabólicas Asociadas (CIBERDEM), Instituto de Salud Carlos III (ISCIII), Madrid, Spain; ^3^ARTIS Development, Las Palmasde Gran Canaria, Spain; ^4^Department of Radiology, Institut De Diagnòstic Per La Imatge (IDI), Hospital Universitari Vall d’Hebron, Barcelona, Spain; ^5^Department of Medicine, Universitat Autònoma de Barcelona, Barcelona, Spain

**Keywords:** ultrasound, rectus femoris, computed tomography, colorectal cancer, body composition

## Abstract

Colorectal cancer (CRC) is a disease with a high prevalence and major impact on global health. Body composition (BC) data are of great importance in the assessment of nutritional status. Ultrasound (US) is an emerging, accessible and non-invasive technique that could be an alternative when it is not feasible to perform computed tomography (CT). The aim of this study is to evaluate the correlation between CT, as a reference technique, and US of the rectus femoris (RF) as a “proof of concept,” in a cohort of patients with CRC and assess the optimisation of results obtained by US when performed by our new semi-automated tool. A single-centre cross-sectional study including 174 patients diagnosed with CRC and undergoing surgery was carried out at the Vall d’Hebron Hospital. We found a strong correlation between CT and US of the RF area (*r* = 0.67; *p* < 0.005). The latter, is able to discriminate patients with worse prognosis in terms of length of hospital stay and discharge destination (AUC-ROC = 0.64, *p* 0.015). These results improve when they are carried out with the automatic tool (area AUC-ROC = 0.73, *p* 0.023), especially when normalised by height and eliminating patients who associate overflow. According to our results, the US could be considered as a valuable alternative for the quantitative assessment of muscle mass when CT is not feasible. These measurements are improved when measuring software is applied, such as “Bat” software.

## 1 Introduction

Muscle plays a fundamental role in the patient’s prognosis and its measurement is now necessary to make a correct nutritional assessment (1). Body composition (BC) evaluation is fundamental for the identification of hidden muscle abnormalities despite adequate, excess or stable weight (2–4). As stated in several recent clinical guidelines, body composition assessment is emphasised as an integral part of daily clinical practise in nutritional assessment (1, 5–7).

In the context of two highly prevalent conditions, such as malnutrition related to the disease (MRD) and sarcopenia, measurement of muscle mass is critical for optimal assessment and identification (1, 7).

Malnutrition related to the disease (MRD) is defined as a subacute or chronic nutritional state in which a combination of varying degrees of over-or undernutrition and inflammatory activity has resulted in altered body composition and reduced function (8, 9).

Sarcopenia is defined as a progressive and generalised loss of skeletal muscle mass, strength and/or physical function, which is associated with an increased risk of adverse outcomes, such as physical disability, poor quality of life, increased mortality (7) and metabolic syndrome (10–14). The presence of sarcopenia at diagnosis or its onset during treatment has been widely associated with poorer outcomes in terms of prognosis, higher rates of surgical complications, poorer response to chemotherapy and greater toxicity, longer hospital stay, and mortality (15–17).

Malnutrition and sarcopenia are very common in patients with oncological pathology (18). Their presence is closely associated with poor clinical outcomes and prognosis. Therefore, the study of BC is important to facilitate diagnoses and allow early intervention (19, 20).

Nowadays, there are several techniques to assess BC and muscle mass, and it is important to recognise the strengths and limitations for an optimal application. Currently, bioelectrical impedance analysis (BIA), muscle ultrasound (US), dual-energy X-ray absorptiometry (DXA), magnetic resonance image (MRI) and computed tomography (CT) are recognised as diagnostic methods for sarcopenia (5, 7).

On the one hand, BIA is a simple, non-invasive, fast and inexpensive method that estimates the BC by measuring the resistance to a low-power alternating current through the body (21). BIA estimates BC as a bicompartmental model: fat free mass (FFM) and fat mass (FM) in kilogrammes and percentage (22). The use of predictive equations is the main limitation of the BIA as it requires a constant hydration and population references that do not always correspond to the clinical reality of the patient (23–25).

Furthermore, DXA is considered to be a precise technique that, provides results from three compartments. It is important to note that since there are only specific attenuation factors for bone and fat, the DXA technique measures only two compartments (bone mass and fat mass) and estimates the third (lean mass) (26, 27). The need for dedicated space for the equipment, trained personnel and exposure to X-ray radiation (even at low doses) are some of the limitations (28).

In addition, US is an emerging accessible bedside, portable, and non-invasive technique as it does not involve ionising radiation for the patient (29, 30). It is able to provide not only quantitative information but also qualitative information by grey scale (31–33). Several skeletal muscle groups have been studied with US, and most of them showed a strong correlation with total muscle mass (32, 34). The quadriceps femoris is the most studied muscle group, especially the rectus femoris (32, 35). Quantitative analysis shows a strong correlation with reference techniques such as MRI or CT (36–38).

CT is emerging as a widely used technique in clinical practise, providing very accurate information for the assessment of BC (39–41). Regional analysis of adipose tissue and muscle at the third lumbar vertebra has been shown to have a high correlation with total BC, and provides significant additional information on tissue quality based on the Hounsfield units (HU) (26, 40, 42).

The assessment of BC by CT image has been widely used in clinical research, especially in those pathologies where CT evaluation is part of the protocol, such as some types of cancer or abdominal pathologies (43). Colorectal cancer (CRC) is a disease of high prevalence and great impact on global health, being the third most commonly diagnosed cancer worldwide and the second leading cause of cancer death (44). Abdominal CT must be routinely performed in colorectal cancer for diagnosis, staging and follow-up (17, 45, 46).

It is crucial to highlight the fluctuations of BC during the evolution of the disease, especially during the active treatment process, which indicates the importance of monitoring its evolution (45, 46). Therefore, an easy to perform and accessible technique is needed to allow us to study BC at any time during the patient’s evolution, taking into account that it does not cause any harm or discomfort to the patient. In this scenario, the US could be a good alternative to CT when the latter is unfeasible to carry out.

In this context, this study is proposed with the aim of assessing: (a) the strength of correlation between the measurements made by ultrasound at the level of the rectus femoris in relation to abdominal CT (as a reference technique) and clinical variables of evolution; (b) to determine which ultrasound variables are significantly associated with clinical evolution; (c) the use of our semi-automated tool would optimise the results obtained by US; (d) whether the use of semi-automatic tools in ultrasound better represents the patient’s clinical reality than manual measurements.

## 2 Materials and methods

### 2.1 Patient selection

We performed a single-centre cross-sectional study including consecutive patients diagnosed with colorectal cancer who underwent oncological surgery at the Vall d’Hebron University Hospital, between May 2021 and September 2023.

The study was approved by the local ethics committee PR (AG) 489/2021 and was conducted in accordance with the Declaration of Helsinki.

All the patients signed the informed consent form before participating in the study. Inclusion criteria: (a) more than 18 years of age; (b) diagnosis of colorectal cancer confirmed by biopsy; (c) patients recruited 48 h after colorectal oncology surgery; (d) acceptance and return of the signed informed consent form signed after clarification of doubts. Exclusion criteria: (a) unable to perform CT scan; (b) unable to undergo ultrasound in the rectus femoris (i.e., amputations); (c) abdominal CT scan performed 30–40 days before recruitment.

### 2.2 Clinical data collection

Patients were recruited 48 h after colorectal oncology surgery. At this time, anthropometric measurements of current weight and height were performed, and anthropometric history (usual weight and weight loss in the previous 6 months) was obtained. Patients’ performance status was assessed using the Eastern Cooperative Oncology Group (ECOG) scale. The remaining demographic and oncological variables were obtained from the clinical history. At the time of recruitment, a tetrapolar bioimpedance was performed using the Bodystat quadscan 4,000.

In this study, we evaluated the potential use of rectus femoris muscle measurement, via ultrasound, as a prognostic criterion for clinical outcomes in patients, specifically considering length of hospital stay and discharge destination.

After discharge, return home from the hospital was considered a natural progression. The need for hospitalisation at home, referral to a social health centre or death was considered an unfavourable outcome with a poor prognosis.

The hospital stay considered favourable in our centre for this type surgery is 3–5 days. Therefore, two hospital stay groups were identified, those with a normal or expected hospital stay (less than 5 days) and those with a prolonged hospital stay (>10 days). The exclusion of patients with stays between 6 and 9 days was a deliberate methodological choice. This decision was informed by the clinical observation that within this range, the reasons for prolonged hospitalisation can vary widely, encompassing both medical complications and non-medical, logistical factors (such as weekend discharge policies, availability of the discharging physician, or unexpected systemic demands on hospital resources). As such, including this group in either the positive or negative outcome categories could introduce significant variability and potential bias into the analysis, undermining the clarity and interpretability of our findings. Our approach aligns with the principle of maximising the interpretability of the study’s outcomes by focusing on patient groups with clear clinical trajectories.

We scored these two prognostic variables together as “good” (good outcome) or “poor” (poor outcome). In this way, those individuals who have a “normal” stay (up to 5 days) and who return home upon discharge are considered to have a good prognosis. This allows for biases such as those patients with a short stay who end up dying.

### 2.3 Rectus femoris ultrasound

Ultrasound measurements of the unilateral right quadriceps rectus femoris (RF) were performed in all patients. Ultrasound imaging and manual measurements were performed by an experienced medical sonographer on the same day of recruitment. A linear portable ultrasound transducer (UProbe L6C Ultrasound Scanner, Guangzhou Sonostar Technologies Co., China) was used, and all the images were acquired with 10 kHz.

**Figure 1:**
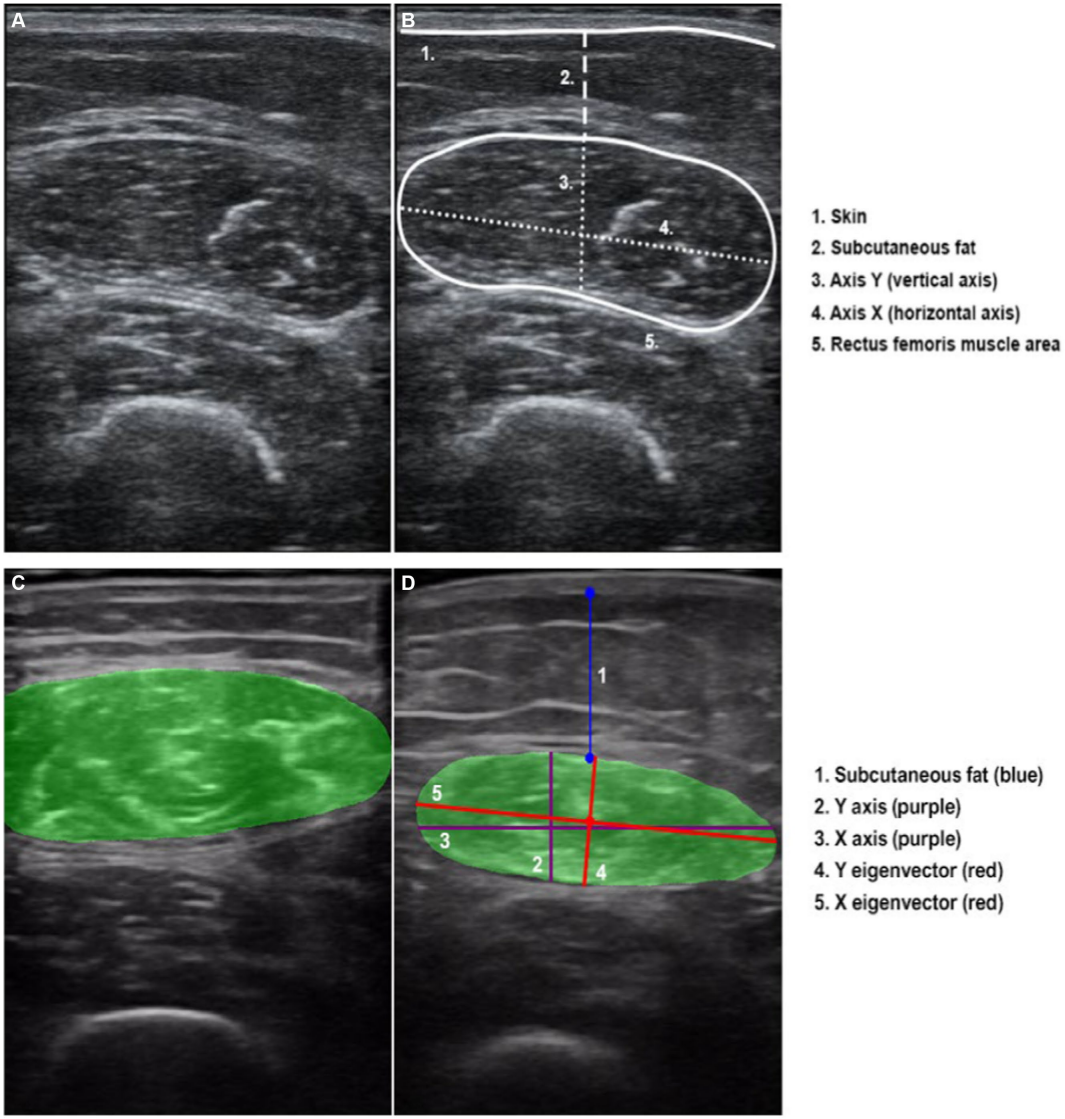
Ultrasound transversal section of rectus femoris. **(A)** Rectus femoris image without overflow. **(B)** Rectus femoris manual measures and scheme of the anatomical structures. **(C)** Image of rectus femoris with overflow. **(D)** Rectus femoris “Bat” measures, representing the “classic *X* and *Y* axes” and the reorientation using the automatic variables of the “*X* and *Y* eigenvector”.

Thigh muscle measurements were performed with the patient in the supine position with knees extended and relaxed. The acquisition site was located two-thirds of the way along the femur length, measured between the anterior superior iliac spine and the upper edge of the patella (29). The transducer was placed perpendicular to the long axis of the thigh with abundant use of contact gel and minimal pressure to avoid muscle compression (Figure 1A).

All parameters were taken as the average of two consecutive measurements in the dominant leg. We took an image in a transversal section, and then measured the cross-sectional area (CSA) in cm^2^, the *X*-axis and *Y*-axis in cm, which corresponded to the linear measurement of the distance between the muscle limits of the rectus femoris (lateral and anteroposterior), and the total fat subcutaneous tissue in cm (Figure 1B). All US parameters were also standardised by the height squared (in cm^2^ for the rectus femoris) (29).

In order to optimise the measurements obtained manually from the US, our group has developed a semi-automatic tool called “Bat.” All the images in crude format (“.dcom”), obtained from the US were first manually marked with the “Bat” tool. Later, the tool generated the same metrics of the RF area obtained manually, adding the grayscale value (0: Black, 255: white) (31, 32).

To analyse these images, a combination of advanced digital techniques, including pixel labelling, Principal Component Analysis (PCA) and centroid identification were used. The latter involves calculating the “centre” of the labelled pixels, similar to determining the equilibrium point of the muscle’s shape within the image. Besides, PCA is also a sophisticated statistical method used to identify patterns in data. Its purpose is to express data in a way that highlights both its similarities and its differences. In simple terms, envisioning the muscle as an ellipse, these axes can be visualised as lines passing through its centre. The first principal axis (major eigenvector) is the direction with the highest variance in the data (pixels) and corresponds to the “longest” diameter of the ellipse. The second principal axis (minor eigenvector) is perpendicular to the first and represents the direction with the next highest variance, corresponding to “shortest” diameter of the ellipse (Figure 1D).

Manual area marking using “Bat” tool was performed by a different researcher to the one who initially measured the image (Figure 1C). It is important to note that both researchers remained blind to any additional clinical or body composition data during both the image acquisition and analysis stages.

In addition, occasionally, the image of the rectus femoris exceeds the dimensions of the transducer, making it impractical to include the entire area in a single image. This circumstance, referred to as “overflow” (Figure 1C), presents a challenge at the image analysis level. When the image has overflow, the area calculation is estimated according to the trajectory of the rectum. As it is not possible to fully analyse the area completely manually or automatically, the level of precision or adjustment of this variable to the reality of the patient is artefactual. Therefore, we categorised the sample into two groups based on the presence or absence of image overflow to assess its potential impact on clinical outcomes.

### 2.4 Computed tomography analysis

Skeletal computed tomography (CT) images focused on the L3 vertebrae were obtained using a multidetector computed tomography scanner (Aquilion Prime SP, Canon Medical Systems, Japan), with the following technical parameters: 135 kV (tube voltage), 1 mm 80 row (detector configuration), tube current modulation, and 0.8 s/rotation (gantry rotation). The following variables were recorded: skeletal muscle mass area or SMA (cm^2^ and %), skeletal muscle mass index or SMI (cm^2^/m^2^), intramuscular adipose tissue area or IMAT (cm^2^ and %), intramuscular adipose tissue index or IIMAT (cm^2^/m^2^), area of visceral fat mass (VFA) (cm^2^ and %), subcutaneous fat (SFA) (cm^2^ and %), visceral fat mass index (VFI) (cm^2^/m^2^), and subcutaneous fat (SFI)(cm^2^/m^2^), and average Hounsfield Units (HU) for each segmented tissue. The CT images centred on the third lumbar vertebra (L3) were analysed using FocusedON-BC software. Tissue quality was assessed based on its average Hounsfield Units (HU) value. Standard thresholds were used as follows: −29 to 150 HU for skeletal muscle, −190 to−30 for subcutaneous adipose tissue and−150 to−50 for visceral adipose tissue (41, 42, 47).

### 2.5 Statistical analyses

Statistical analyse were performed using Python 3. Continuous variables are presented as mean ± standard deviation (SD) for normally distributed variables and median ± interquartile range (IQR) for non-normally distributed variables. Categorical variables are presented as percentages. Statistical significance was accepted at *p* < 0.05.

To assess whether a given numerical variable can be used as a predictive criterion for a patient’s clinical outcome, the separability of groups associated with treatment success or failure was assessed. Treatment success or failure was determined on the basis of several clinical variables, such as length of hospital stay or discharge destination. Group separability was assessed using the Student’s t-test when the two groups had a normal distribution and equivalent variances. The Mann–Whitney U test was used when neither condition applied. The Anderson-Darling method was used to evaluate the normality of the distribution of the numerical variables. The Levene’s test was used to confirm the equivalence of variances.

In addition, ROC curves were used to quantify the overall precision of each method by measuring the area under the curve (AUC).

Given the innovative nature of the methodology being tested, our study was designed as a proof-of-concept (PoC) investigation. Primarily to explore the feasibility and potential efficacy of this new approach, rather than to provide definitive evidence of its effectiveness. This inherent uncertainty in the expected outcomes and effect sizes made traditional sample size calculation methods challenging to apply effectively. In this context, the sample size for our PoC study was determined based on practical considerations, including the availability of subjects and resources, with an emphasis on obtaining a preliminary assessment of the methodology’s feasibility and potential signals of effectiveness.

Moreover, various graphs and images were generated to better illustrate the statistical results.

## 3 Results

### 3.1 Study population

We recruited 174 patients (see Table 1), predominantly male, with a mean age of 68.91 ± 11.52 years old. All participants had colorectal neoplasia, with the colon and sigmoid colon being the most commonly affected sites. Notably, 65% of the recruited patients presented with stage II-III disease at the time of diagnosis, although an overwhelming 95% maintained a good baseline functional status (ECOG≤1). According to the GLIM criteria (assuming that all patients have an etiologic criteria + at least 1 phenotypic criteria including weight loss >5% during the last 6 months, BMI < 20 kg/m2 if <70 years old or < 22 kg/m2 if >70 years old and a reduction in muscle mass measured by BIA), 21% of the patients met the criteria for malnutrition, although the average BMI was above the normal range (BMI 26 kg/m2) (5). Sarcopenia was screened screening using the SARC-F questionnaire, which showed a 9% risk of sarcopenia within in the sample.

**Table 1:** Patients’ demographic, clinical and anthropometric characteristics.

Characteristics	Study population (*n* = 174)
Sex	
Female	64 (37%)
Male	105 (60%)
Age (years)	68.91 ± 11.52
Tumour location
Colon	78 (45%)
Sigmoid	53 (30%)
Rectum	23 (13%)
Cecum	15 (9%)
Anus	1 (1%)
TNM stage	
I	31 (18%)
II	54 (31%)
III	60 (34%)
IV	10 (6%)
ECOG	
0	141 (81%)
1	25 (14%)
2	4 (2%)
3	1 (1%)
4	1 (1%)
BMI (kg/m^2^)	26.27 ± 4.61
Malnutrition by GLIM criteria	36 (21%)
Suspicion sarcopenia by SARC-F	16 (9%)
Ultrasound RF area (cm^2^)	3.89 ± 1.35
CT muscle area – SMA (cm^2^)	112.59 ± 28.52
Discharge destination	
Home	160 (92%)
No home	14 (8%)
Length hospital stay (days)	7.72 ± 10.12
≤ 5 (*n* = 112)	3.56 ± 0.78
≥ 10 (*n* = 31)	22.35 ± 15.44

BMI, body mass index; GLIM: Global leadership Initiative of Malnutrition, ECOG, Eastern Cooperative Oncology Group scale; RF, rectus femoris; CT, computed tomography; SMA, skeletal muscle area.

### 3.2 Quantifying muscle mass: US vs. CT

**Figure 2:**
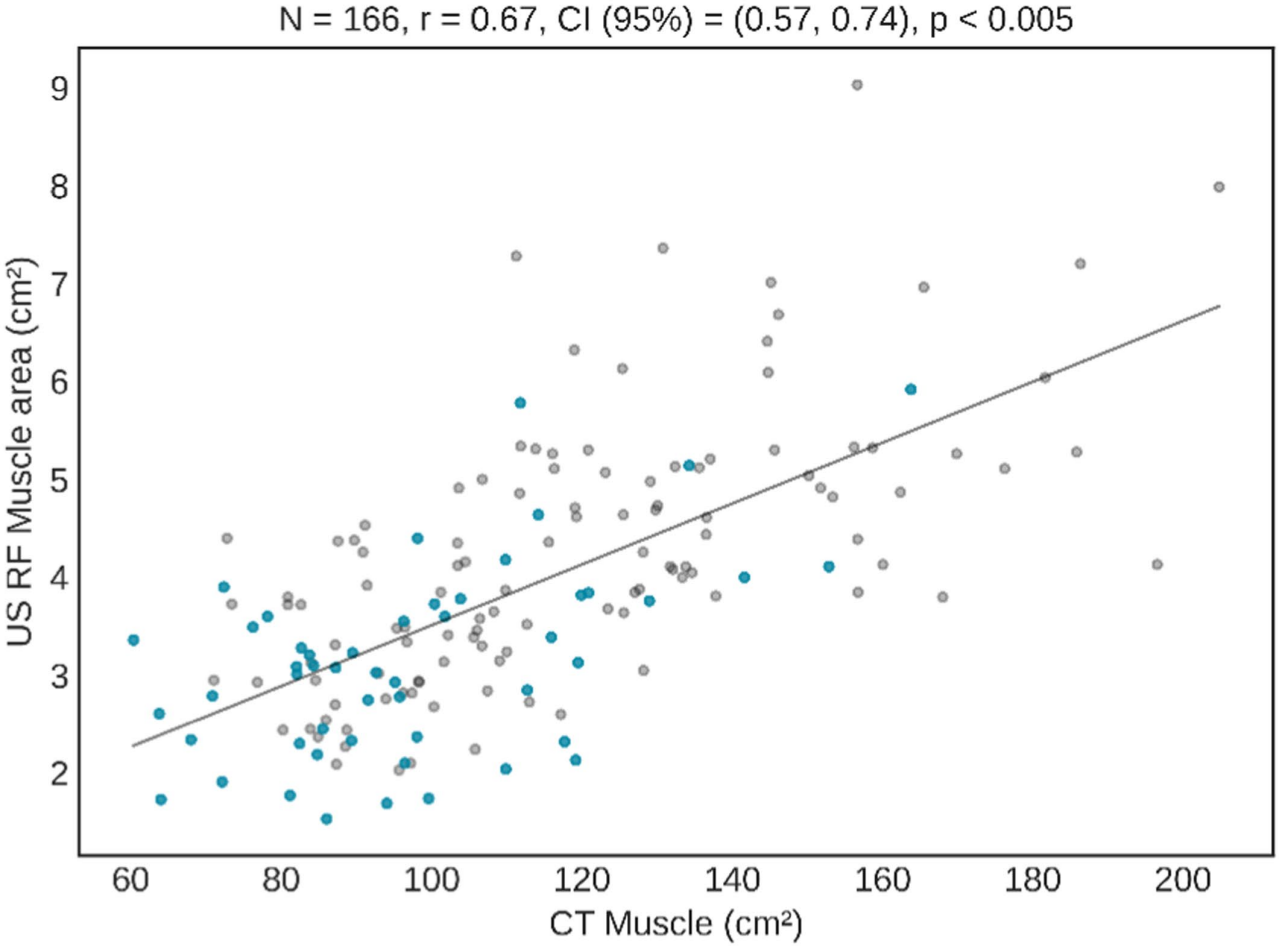
Correlation between CT muscle area and US RF area. Blue dots represent subjects with overflow in US image, and grey dots patients without overflow.

The amount of muscle measured by the rectus femoris present a strong and statistically significant correlation with the results of the CT muscle area, especially in the variables “*Y* axis” and “area” (Table 2). In Figure 2 a graphical representation illustrates the correlation between the results of the RF ultrasound area and the results of the abdominal CT muscle area. The graphic presentation clearly shows a remarkable correlation between both CT and US findings. In particular, patients without overflow (represented by blue dots) are closer to 0, indicating a smaller muscle mass. The graph also highlights a noticeable clustering of the samples within a narrower numerical range, which represents a challenge in separating patients based on their clinical evolution.

**Table 2:** Correlation between CT muscle area and US RF variables (area, *X* and *Y* axis).

CT variable	US variable	*N*	*R* ^2^	IC (95%)	*p* value
CT Muscle area (cm^2^)	US RF Muscle area (cm^2^)	166	0.67	(0.57, 0.74)	*p* < 0.005
CT Muscle area (cm^2^)	US RF Muscle X (cm)	166	0.53	(0.41, 0.63)	*p* < 0.005
CT Muscle area (cm^2^)	US RF Muscle Y (cm)	166	0.66	(0.56, 0.74)	*p* < 0.005

CT, computed tomography; US, ultrasound; RF, rectus femoris.

### 3.3 US quantification of muscle mass and clinical evolution

The averages of the main variables obtained by ultrasound have been obtained and presented in Table 3. It can be seen that the possibility of having a good or bad prognosis is related to the manual area (*p* = 0.041), and especially when we normalise this measurement by the square of the height (m^2^), improving the ability to predict the patient’s outcome. In addition, other manual and automatic variables also improve their prognosis capabilities when normalised by patient’s height. In example, muscle area improves its AUC from 0.62 to 0.64 and *Y* axis from 0.59 to 0.61.

**Table 3:** Clinical evolution using US RF variables using all the image, and separating those with and without overflow, normalised or not by height.

	With overflow	Unnormalized by height						Normalised by height (/m^2^)					
Manual metrics	US variable	All (*n* = 143)	Good outcomes (*n* = 111)	Poor outcomes (*n* = 32)	*p* value	ROC (AUC)	SE	All (*n* = 143)	Good outcomes (*n* = 111)	Poor outcomes (*n* = 32)	*p* value	ROC (AUC)	SE
	US RF muscle area (cm^2^)	3.92	4.06	3.45	0.041	0.62	0.06	1.4	1.45	1.22	0.015	0.64	0.05
	US RF muscle x (cm)	3.65	3.67	3.56	0.315	0.56	0.06	1.32	1.33	1.28	0.095	0.57	0.06
	US RF muscle y (cm)	1.32	1.35	1.24	0.087	0.59	0.06	0.47	0.49	0.44	0.064	0.61	0.05
	Without overflow	Unnormalized by height	Normalised by height (/m^2^)	US variable	All (*n* = 42)	Good outcomes (*n* = 33)	Poor outcomes (*n* = 9)	*p* value	ROC (AUC)	SE	All (*n* = 42)	Good outcomes (*n* = 33)	Poor outcomes (*n* = 9)	*p* value	ROC (AUC)	SE	
US RF muscle area (cm^2^)	3.12	3.24	2.7	0.178	0.65	0.1	1.16	1.21	0.99	0.099	0.71	0.1
US RF muscle x (cm)	3.32	3.37	3.14	0.19	0.68	0.1	1.25	1.28	1.15	0.065	0.68	0.12
US RF muscle y (cm)	1.18	1.2	1.13	0.522	0.55	0.11	0.44	0.45	0.41	0.248	0.56	0.1
Software metrics	US RF muscle area-s (cm^2^)	3	3.12	2.52	0.061	0.7	0.1	1.12	1.17	0.93	0.023	0.73	0.1
	US RF muscle y-s (cm^2^)	1.15	1.18	1.04	0.152	0.66	0.09	0.43	0.44	0.38	0.053	0.68	0.09
	US RF x-eigenvector (cm)	3.29	3.33	3.16	0.374	0.6	0.13	1.24	1.26	1.16	0.253	0.59	0.14
	US RF Muscle y-eigenvector (cm)	1.16	1.19	1.03	0.085	0.69	0.08	0.43	0.45	0.38	0.024	0.72	0.09

US, ultrasound; RF, rectus femoris; SE, standard error.

Similarly, when the patient’s whit overflow was removed from the sample, muscle area improves its AUC from 0.64 to 0.71.

**Figure 3:**
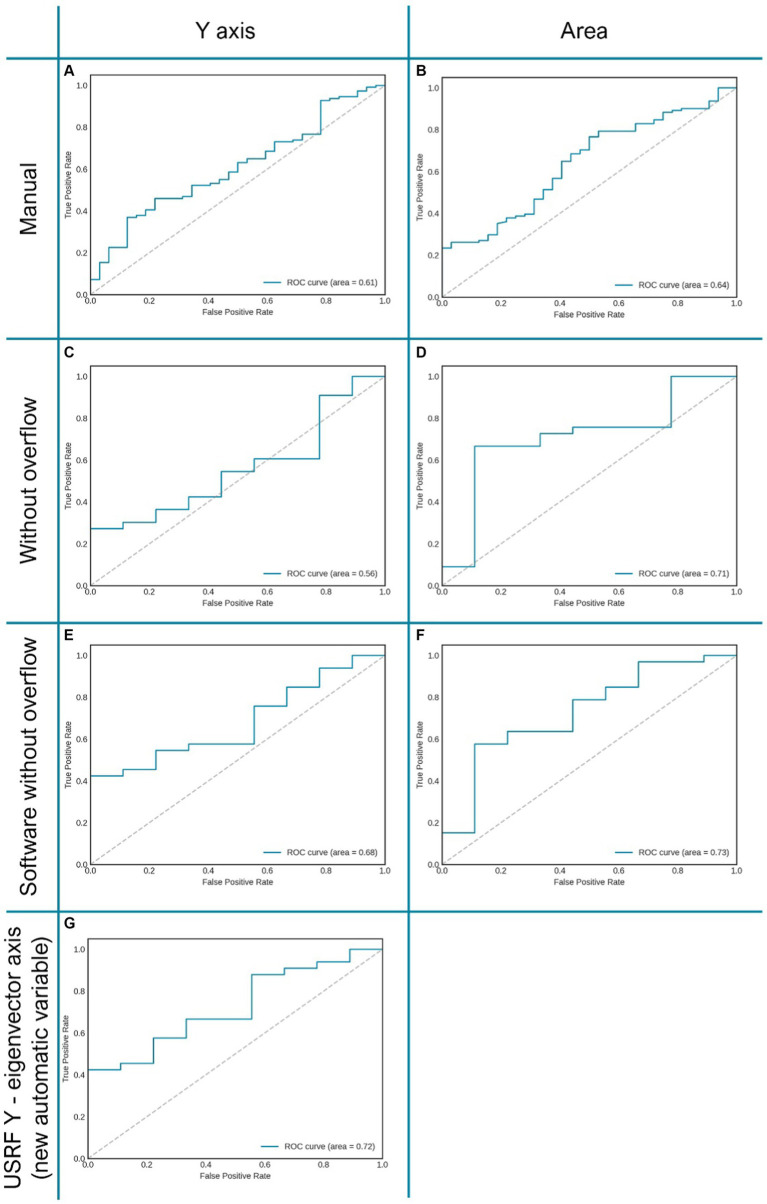
Graphic representation of several AUC of ROC curves of the variables “*Y* axis” and “area” related to clinical prognosis. Allows assessing the improvement of the same variable when changing the measurement method (manual or automatic). **(A,B)** RF area and “*Y* axis” measured manually; **(C,D)** RF “*Y* axis” and area measured manually in patients without overflow. **(E,F)** RF “*Y* axis” andarea measured automatically with “Bat” tool in patients without overflow. **(G)** “Eigenvector *Y* axis,” proposed vector obtained automatically through image analysis with the “BAT tool”.

On the other hand, the use the software tool, which allows carrying the analysis in a more automatic and user independent way, also increases the performance of the different metrics. For instance, muscle *Y* axis improves from 0.56 to 0.68, and even to 0.72 when using the y-eigenvector. Similarly, muscle area improves from 0.71 (after normalised by height and remove overflow) to 0.73 using semiautomatic tool. This improvement can be observed in the ROC curves displayed in Figure 3.

Although this trend can be clearly seen in the results, the difference between the manual and automatic metrics is not statistically significant (*p*-value >0.05).

## 4 Discussion

This study is, to our knowledge, the first to use a semi-automatic tool in the evaluation of muscle ultrasound (US) in rectus femoris.

US has gained widespread acceptance and is nowadays included in several influential body composition guidelines (1, 7, 48). Positioned as the “stethoscope of body composition,” US is valued for its accessibility, cost-effectiveness, and bedside applicability (49). In our study, measurement of muscle mass using US at the RF showed a robust correlation when compared to quantification using CT as the reference technique (*r* = 0.67, IC 0.57–0.74, *p* < 0.001). These results are similar or superior to those reported in other studies, such as Paris et al. shows (*r* = 0.45, *p* < 0.01) or Lambell et al. (0.7, *p* < 0.01) (50, 51). However, the paucity of published evidence comparing ultrasound with CT is probably due to the logical challenge of synchronising both tests at clinically comparable times.

Taking into account the previously presented information, patients with colorectal cancer emerge as particularly suitable candidates, as they require CT scans as part of their follow-up, staging and overall assessment (45, 52). This positioning allows CT to be used as an “opportunistic” technique to analyse BC with a high degree of precision, thus providing a valuable validation platform for emerging techniques, such as US (53, 54). The results derived from this study contribute significantly to our understanding, endorsing US as a technique with acceptable results for screening and diagnosis of muscle alterations, validated against CT, in patients with colorectal cancer. Thus, it would be an alternative tool for use in the future when TC is not feasible.

It should be noted that muscle ultrasound is a technique that provides us quantitative (area and *Y* axis) and qualitative muscle information (grey scale) (29). This information is obtained directly from muscle mass and has been shown to have a very good correlation with the patient’s functional capacity and prognosis (55, 56). In the case of bioimpedance, a doubly indirect method of assessing body composition, quantitative information on muscle mass can be obtained through fat free mass, but not qualitative information on muscle mass (22). The phase angle is a parameter that we can obtain from the BIA that has a highly impactful prognostic value (57, 61). However, it is a parameter that comes from a complete body evaluation, not from muscle mass in particular. On the other hand, DXA is a highly accurate technique for evaluating body composition, but it is not a particularly good technique for evaluating muscle status since it does not provide information on muscle quality (26). Furthermore, it is an expensive and inaccessible technique.

Reduction in muscle mass (MM) is strongly associated with a prognosis in terms of postoperative complications, prolonged hospital stays, discharge outcomes, treatment response and mortality (61). Consistent with this premise, our study results confirm that lower MM, as measured by US, associated with longer hospital stays and decreased likelihood of discharge home, essentially indicating a more difficult prognosis for the patient. Given the accessibility of US as a technique, serious consideration should be given to its more frequent incorporation into protocols for prevention and clinical optimization in patients with colorectal cancer. Furthermore, the results obtained can play a key role in tailoring multimodal treatments in cases where low muscle mass is evident or its deterioration is observed over time.

On the other hand, one of the weaknesses of muscle ultrasound is its operator-dependent nature, requiring skilled personnel to perform it accurately (39, 59, 60). It is also necessary to implement standardised protocols so that their results are comparable (32). A key factor contributing to the variability of findings is the manual nature of quantitative measurements, such as the area or the X and Y axes, which are subject to the operator interpretation. There are various software programmes that allows us to obtain the grey scale through manual measurements, but currently, there are no tools for the rest of the ultrasound metrics at the RF level that are automatic (31).

In this study, we begin to develop a near automatic tool that simplifies the process by requiring only the manual marking of the RF area. This innovative approach facilitates the automatic derivation of other variables from the initial manual marking. Several processes in medicine have shown that the automation of measurements and the use of software that reduces the intervention of the “researcher’s hand” is superior and optimises results (61–63). When performing ultrasound imaging, for example of the rectus femoris muscle, there’s a common challenge related to the orientation of the ultrasound probe. Even a slight rotation or tilt of the probe can alter the appearance of the muscle in the image. However, the described method, based on principal axes (eigenvectors), significantly mitigates this problem by incorporating principal component analysis (PCA) to identify the principal axes of the muscle image effectively addresses this challenge. Regardless of the probe’s orientation, the principal axes of the muscle’s image will consistently adjust. This means that the major and minor axes of the ellipse representing the muscle are consistently aligned with the directions of maximum variance in the image, regardless of how the ultrasound probe is held.

This technique featly reduces the reliance on the operator skill or consistency in probe placement. Different observers can perform the scan, and the main axes will remain consistent for the same anatomical structure, ensuring more objective and reproducible measurements. By focusing on the intrinsic geometric properties of the muscle tissue, as represented by the ellipse in the image, measurements become more reflective of the actual dimensions of the muscle and less dependent on the probe positioning. Consequently, this approach leads to more accurate and objective assessments of muscle size, shape, and potentially its health status.

Firstly, we carefully analysed the correlation between our measurements and those performed manually, and found robust and statistically significant associations for all variables (refer to Table 3). RF area (cm^2^) emerged as the variable with the strongest correlation with CT, although results were also noted for the *Y* axis (see Table 2). This may be related to the fact that the favourable performance of the axis due to its vertical measurement, which is not affected by possible image displacement from the screen. In this sense, we observed that a significant percentage of the images (62%) acquired according to our protocol in the lower third of the leg showed an area that extended beyond the edges of the screen. This overflow situation, where the area extends beyond the edges of the screen, introduces a potential source of imprecision in area measurements compared to situations where the area is fully displayed. We therefore, stratified the sample into two groups, with and without overflow. We re-run the clinical correlation in the group without overflow, and observed an improvement in the ROC curves, indicating a lower rate of false positives and false negatives. An increase in the correlation with clinical complications, discharge destination and hospital stay were also observed. However, it is recognised that these results may be influenced by the fact that many patients without overflow generally have worst muscle mass.

A limitation of our tool is its partial automation, which requires a manual measurement by a researcher. However, it has been shown that a reduction in manual measurement leads to significantly better results. It is necessary to carry out studies with a larger sample size to fully automate the tool. In addition, it would have been interesting to measure the RF area a few centimetres closer to the patella to see if this would provide an improvement that we should definitely include in our protocols when eliminating the overflow. Furthermore, assessing the usefulness of these measurements in patient follow-up and exploring other prognostic variables such as post-operative complications or mortality is an interesting avenue for further research. Our ongoing studies are designed to a comprehensively address these issues.

## 5 Conclusion

In conclusion, the significant correlation levels observed in our study prompt led to consider the US as an alternative for the quantitative assessment of muscle mass when CT is not feasible. It is noteworthy to highlight the pioneering role of “Bat,” the first software to allow semi-automatic derivation of metrics in rectus femoris measurements. The application of principal axis analysis in ultrasound imaging is proving to be a powerful approach to standardising muscle tissue measurements. This methodology effectively overcomes the challenges associated with variable probe orientation, thereby improving the accuracy, objectivity, and reproducibility of measurements. Such improvements are of paramount importance in clinical settings, contributing significantly to accurate diagnosis and monitoring. In addition, the approach reduces inter and intra-observer dependencies, further enhancing the precision and objectivity of the results. This not only improves their separability but also partially compensates for one of the recognised weaknesses of muscle ultrasound.

Whilst acknowledging these advances, it is prudent to emphasise the need for additional studies to fully automate the “Bat” tool and thoroughly reassess its clinical utility. This ongoing research is critical for refining and optimising muscle assessment methodologies, and ensuring continued progress in the field.

## Data availability statement

The original contributions presented in the study are included in the article/supplementary material, further inquiries can be directed to the corresponding authors.

## Ethics statement

The studies involving humans were approved by Comité de Ética de la Investigación con medicamentos del Instituto de Investigación Vall d’Hebron. The studies were conducted in accordance with the local legislation and institutional requirements. The participants provided their written informed consent to participate in this study.

## Author contributions

FP: Conceptualization, Methodology, Supervision, Writing – original draft, Writing – review & editing, Validation. FM: Investigation, Writing – review & editing. MR: Investigation, Writing – review & editing. AL: Investigation, Writing – review & editing. AZ: Investigation, Writing – review & editing. JM: Investigation, Software, Writing – review & editing. RG: Formal analysis, Software, Writing – review & editing. AR: Resources, Writing – review & editing. NR: Resources, Writing – review & editing. AC: Writing – review & editing. RB: Conceptualization, Funding acquisition, Writing – original draft, Writing – review & editing.
